# STAKEHOLDER PERSPECTIVES ON DESIGN, IMPLEMENTATION, AND SUSTAINMENT OF SERVICES FOR OLDER VETERANS

**DOI:** 10.1093/geroni/igz038.2772

**Published:** 2019-11-08

**Authors:** Samantha Solimeo, Bret Hicken

**Affiliations:** 1 Center for Access and Delivery Research and Evaluation Primary Care Analytics Team Iowa City VA Health Care System, Iowa City, Iowa, United States; 2 Veterans Rural Health Resource Center-SLC, Salt Lake City, Utah, United States

## Abstract

A majority of United States Veterans are older adults, compelling healthcare systems such as Veterans Health Administration to attend to their unique needs when designing and implementing programs for workforce development and service delivery. In this symposium authors will present findings from four studies examining how older Veterans’ needs and preferences affect implementation and sustainment in a variety of settings. Presenters demonstrate how: 1) understanding Veterans’ perspectives and preferences for measuring functional status may inform the improvement of care coordination in the primary care setting; 2) the role of population characteristics in implementation of geriatric patient centered medical home teams (i.e., GeriPACTs); 3) the interaction of patient, provider, and delivery system information needs in limiting sustainment of diverse initiatives to improve osteoporosis screening and management for Veterans; and 4) the factors affecting transferability and sustainment of rural and geriatrics-focused quality improvement initiatives beyond local settings. Beyond their focus on how older adults’ needs are reflected or shape implementation, the studies illustrate the application of qualitative data to clinical practice and workforce development.

